# Waveguide photoreactor enhances solar fuels photon utilization towards maximal optoelectronic – photocatalytic synergy

**DOI:** 10.1038/s41467-020-20613-2

**Published:** 2021-01-15

**Authors:** Joel Y. Y. Loh, Abhinav Mohan, Andrew G. Flood, Geoffery A. Ozin, Nazir P. Kherani

**Affiliations:** 1 Department of Electrical and Computing Engineering, University of Toronto, 10 King’s College Road, Toronto, ON M5S 3G4 Canada; 2 Department of Chemistry, University of Toronto, 80 St. George Street, Toronto, ON M5S 3H6 Canada; 3 Department of Materials Science and Engineering, University of Toronto, 140 College Street, Toronto, ON M5S 3E4 Canada

**Keywords:** Pollution remediation, Photocatalysis, Chemical engineering, Optical manipulation and tweezers

## Abstract

A conventional light management approach on a photo-catalyst is to concentrate photo-intensity to enhance the catalytic rate. We present a counter-intuitive approach where light intensity is distributed below the electronic photo-saturation limit under the principle of light maximization. By operating below the saturation point of the photo-intensity induced hydroxide growth under reactant gaseous H_2_+CO_2_ atmosphere, a coating of defect engineered In_2_O_3-*x*_(OH)_*y*_ nanorod Reverse Water Gas Shift solar-fuel catalyst on an optical waveguide outperforms a coated plane by a factor of 2.2. Further, light distribution along the length of the waveguide increases optical pathlengths of the weakly absorptive green and yellow wavelengths, which increases CO product rate by a factor of 8.1-8.7 in the visible. Synergistically pairing with thinly doped silicon on the waveguide enhances the CO production rate by 27% over the visible. In addition, the persistent photoconductivity behavior of the In_2_O_3-*x*_(OH)_*y*_ system enables CO production at a comparable rate for 2 h after turning off photo-illumination, enhancing yield with 44-62% over thermal only yield. The practical utility of persistent photocatalysis was demonstrated through outdoor solar concentrator tests, which after a day-and-night cycle showed CO yield increase of 19% over a day-light only period.

## Abstract (web summary)

Concentrating photo-intensities on photocatalyst has diminishing returns. Here the authors show the catalyst on glass rod waveguide at optimal low intensity results in high efficiency in the gas phase reverse water gas shift reaction in an annular glass cylindrical rod photoreactor.

## Introduction

Displacing the fossil fuel industry with the goal of climate change mitigation and environmental preservation requires a carbon-neutral solar fuels catalysis cycle from product to reactants and vice versa. The conversion of solar radiation to solar fuels such as carbon monoxide to be further processed as methanol^1^ is a thermodynamically more feasible and potentially high yielding^2^ than direct CO_2_ hydrogenation^3–5^.

However, the conventional tendency in order to maximize a photo-catalyst product rate is to concentrate the incident light intensity with the assumption that the reaction rate is dependent on the density of excited charge carriers^6,7^. Since the volumetric photo-generated electron–hole density far exceeds the possible concentration of surface active sites, photon-to-charge-to-yield efficiencies are poor at high photon intensities due to increasingly high rates of recombination processes^8^, which results in a semi-saturation limit^9^. The contradiction between achieving high photon-to-yield efficiencies (for maximizing the size of solar coverage) and high product rate (for maximizing the yield per catalyst mass or area) requires light to a catalyst management approach that encompasses both optical and charges based catalysis considerations^10^.

Herein, we demonstrate the principle of light maximization by performing the gas phase reverse water gas shift (RWGS) reaction (CO_2_ + H_2_ → H_2_O + CO)^11–14^ in an annular glass cylindrical rod photoreactor^15–17^ with optimized uniformity and thickness of a photo-charge driven In_2_O_3−*x*_(OH)_*y*_ nanorod coating^18–21^. We highlight an important advantage that has to the best of our knowledge been overlooked in similar waveguide studies, which is overcoming both the photo-current and product semisaturation limit at high photo-intensities. This improves photon-to-yield efficiencies. Further, the omni-directional optical scattering of the nanorod coatings enables light propagation due to their antenna-like geometry. Along with internal reflectivity inside the photo-reactor, it is possible to internally illuminate the entirety of the photo-catalyst coating. In addition to that, multiple reflection pathways of the weakly absorptive green and red wavelengths enable the band-gap trap states of the photo-catalyst to be populated^22^ and compel excited charge carriers from valence and conduction band edges to relax through the surface catalyst pathway rather than through bulk recombination^23–25^.

## Results

### Photo intensity trends

**Fig. 1:**
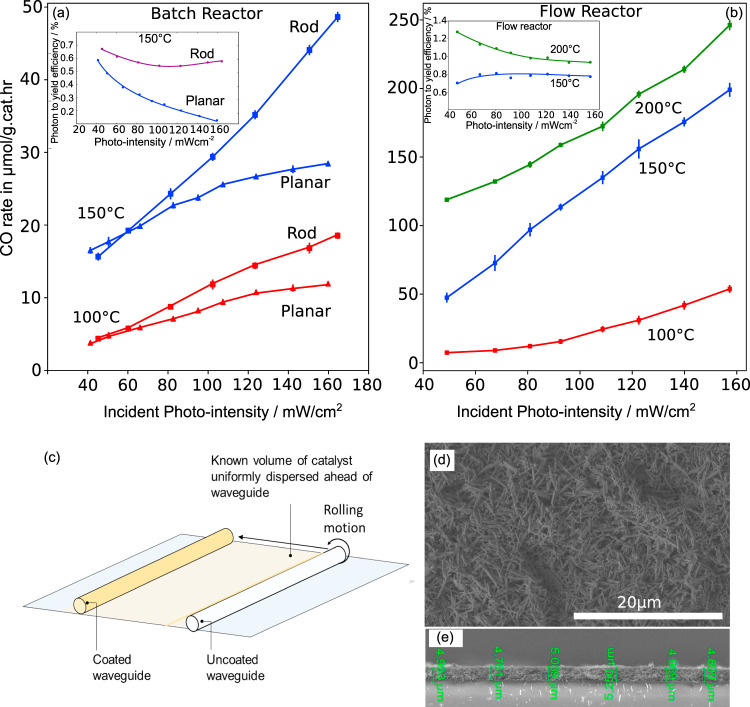
Batch and flow reactor results. **a**, **b** The CO production rates and associated activation energies and photon to yield quantum efficiencies for the batch reactor and flow reactor configurations, respectively. (a) Shows the CO rates for the coated cylindrical rod waveguide and coated planar substrate at 100 and 150 °C. The coated planar CO rates at 150 °C showed semi-saturation trends at increasing photo-intensities, while the coated rod showed a linear increase. Insets of (**a**) shows the photon to yield efficiencies of the coated planar and rod geometries at 150 °C batch reactor temperature with increasing photo-intensities, indicating that the coated rod waveguide has a more consistent efficiency of 0.8–0.9%. **b** The CO rates for the coated rod waveguide at 100, 150, and 200 °C flow reactor temperatures. Inset to (**b**) shows that the coated waveguide rods in the flow reactor have consistent photon to yield efficiencies with increasing photo-intensities. **c** Coating procedure of the quartz rod using a thin film colloidal solution of In_2_O_3−*x*_(OH)_*y*_ nanorods. **d**, **e** The SEM topographic and cross-sectional view of the nanorod coating.

In this section, we compare trends in the CO production rate, activation energies, and photon to yield efficiencies for the coated planar and waveguide cylindrical rod geometries under batch reactor testing performed at different photo intensities and temperatures. The coating thickness of the indium oxide hydroxide (In_2_O_3−*x*_(OH)_*y*_) nanorod coatings on both surfaces are 5 ± 0.3 µm each. The nanorods comprise arrays of necked nanoparticles 10–20 nm in diameter, which enables photo-generated charges to drift within the nanorod structure and enable injection of the charges into catalytic sites. The coated waveguide (diameter of 5 mm, length of 6 cm) is encapsulated in grease-free aluminum foil (Kurt J. Lesker, UHV Al foil, #FOILA24) along the length and back end of the waveguide to maximize side and backpropagation. Figure 1a shows that the CO batch reactor production rate for the coated planar substrate at 150 °C increases to 20 µmol/g.cat.hr for corresponding photo-intensities ranging below 65 mW/cm^2^, and more slowly to 27 µmol/g.cat.hr at corresponding photo-intensities above 81 mW/cm^2^. The diminishing rate of increase of the CO production rate is mirrored in the corresponding decrease in CO quantum efficiency (0.6–0.2%)^26^, Fig. 1a inset. Conversely, the rate of increase of the CO production rate for the coated waveguide at 150 °C does not decrease at higher incident photo-intensities and instead presents a fairly linear rate of increase and correspondingly a relatively constant quantum efficiency (0.6–0.7%). At 200 °C, the rates of the coated rod also increase (Supplementary Fig. 1) albeit more slowly with increasing photo-intensities in the same fashion as that for the coated planar substrate, however, its photon to yield efficiency is generally greater than the coated planar substrate by an additional absolute 0.25% at all values of incident photo-intensities (Supplementary Fig. 2). In general, the batch reactor rates with increasing temperatures show that the Al encapsulated coated waveguide cylindrical rod is more productive than the planar coated geometries at photo-intensities greater than 60 mW/cm^2^.

The apparent activation energies at an incident photo-intensity range of 120–130 mW/cm^2^ (determined from the averaged rates as a function of temperature) are 23.5 and 25.6 kJ/mol for the two geometries (waveguide rod and planar, respectively) (Supplementary Fig. 3a). The difference in the activation energies between the encapsulated and un-encapsulated coated rod waveguides is fairly minor at 2.99 kJ/mol, and the CO yield for the aluminum encapsulated but uncoated rod waveguide is less than 2% of that of the coated rod waveguide, showing that the aluminum foil encapsulating the uncoated rod does not contribute chemically to the reaction rate. The apparent activation energies under dark conditions for the coated planar and encapsulated coated rod waveguide geometries are fairly similar at 60.0 and 62.1 kJ/mol, respectively. There is therefore a larger difference in the increase in activation energies between the dark and photo-illuminated coated planar/waveguide samples.

We now turn to gauge the performance of a flow photoreactor with a single coated In_2_O_3−*x*_(OH)_*y*_ glass rod operating at a flow rate of 5 sccm for H_2_ and 1 sccm for CO_2_ (Fig. 1b). It is found that the CO production rate under thermal only dark conditions increases exponentially as per the Arrhenius equation to 108.3 µmol/g.cat.hr corresponding to a temperature increase from 100 to 200 °C (Supplementary Fig. 3b). This implies a flow-reactor associated activation energy of 48.1 kJ/mol in the dark, which decreases significantly to 23.8 kJ/mol under a photo-illumination intensity of 139 mW/cm^2^. With increasing photo-intensities at the flow reactor temperature of 200 °C, the CO rate increases significantly from 118.9 to 246.2 µmol/g.cat.hr. The photon to yield efficiency with varying photo-intensities is more consistent than that of the coated planar substrate, with efficiencies of 0.7% at high photo-intensities and 150 °C. These results show that back illumination of the catalyst coating through the internal optical reflection in a quartz waveguide, as well as the high reactant gas residence time in a flow reactor configuration, result overall in high quantum efficiencies over the photo-intensity range.

### Electrical and photo-spectroscopy results

**Fig. 2:**
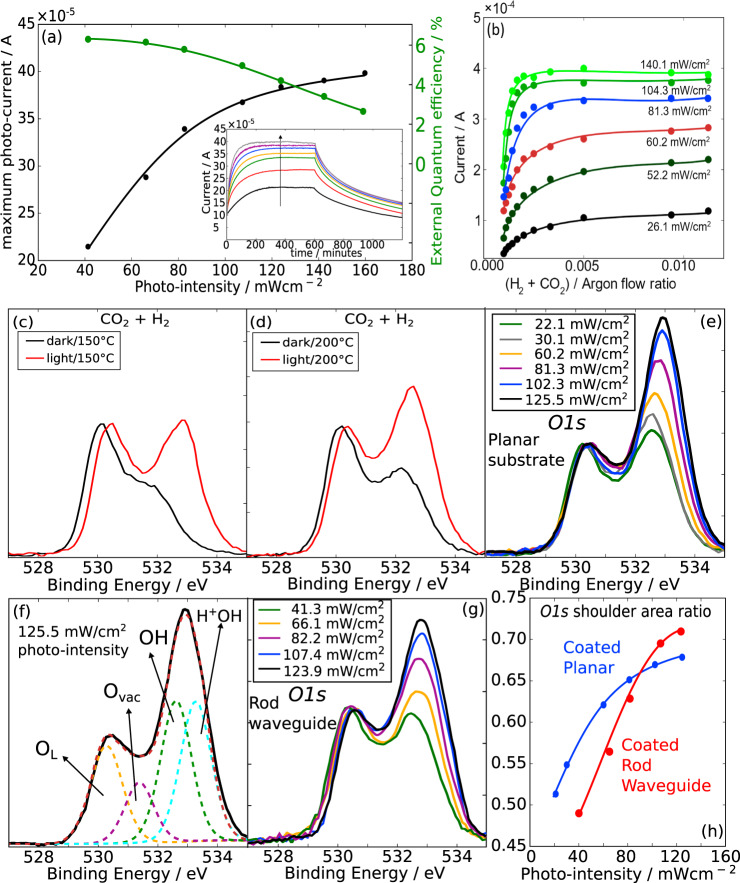
Relating photo-charge and surface reactivity with photo-intensities. The photo-current trend with increasing photo-intensities; photocurrent changes with increasing flow concentration of 1 sccm of H_2_ and 1 sccm of CO_2_ in argon; glovebox protected XPS measurements of In_2_O_3−*x*_(OH)_*y*_ nanorods coated on planar and cylindrical rod geometries after reactions in dark and photo-illuminated conditions at 1:1 H_2_:CO_2_ pressure ratio atmosphere at various temperatures. **a** The photocurrent trend of the coated planar substrate under increasing photo-intensities shows a sharp rise at low photo-intensities below 100 mW/cm^2^ and semi-saturation at higher photo-intensities. **b** Photocurrent increase with increasing H_2_ + CO_2_ gas concentrations and with increasing photo-intensities. **c**, **d** The *O1s* spectra of In_2_O_3−*x*_(OH)_*y*_ nanorods on a planar surface at 150 °C and 200 °C post dark and photo-illumination of one hour in H_2_ + CO_2_ atmosphere (inset). The *O1s* spectrum of In_2_O_3−*x*_(OH)_*y*_ nanorods as prepared on a planar surface, after an hour in a vacuum. The species with FWHM widths of less than 1.3 eV are oxide lattice, oxygen vacancy, and OH groups. **e** *O1s* spectra under H_2_ + CO_2_ at 200 °C under increasing photo-intensities of 1 h. The OH shoulder increases significantly with increasing photo-intensities. **f** The oxide species of lattice oxide, oxygen vacancies, OH, and H^+^OH species are seen for the sample under the highest photo-intensity of 125.5 mW/cm^2^. **g** The *O1s* spectra of the In_2_O_3−*x*_(OH)_*y*_ nanorods coated on a rod waveguide after photo-illumination at 200 °C for 1 h, under increasing photo-intensities. **h** The *O1s* shoulder ratio of the In_2_O_3−*x*_(OH)_*y*_ rods on planar substrates and rod waveguides as a function of increasing photo-intensities.

We discuss the possible rationale behind the aforementioned optical waveguide reactor performance, with photo-current measurements under varying photo-intensities and under varying reactant gas concentrations. While the obvious factor of poor photo-absorption by the subsurface may seem to account for the difference in the CO production yield of the waveguide geometry over the planar substrate, it does not fully account for the relatively stable photon to yield efficiency with increasing incident photo-intensities for the coated waveguide and a lower photon to yield efficiency for the planar substrate at high incident photo-intensities. This indicates that the distribution of the incident photo-illumination over a larger surface area due to the waveguide geometry plays a significant role in the higher CO production rate. As Fig. 2a shows the generated photocurrent of an In_2_O_3−*x*_(OH)_*y*_ coated planar substrate as a function of photo-intensities rises sharply below ~60 mW/cm^2^ but slowly increases beyond a higher photo-intensity of 81 mW/cm^2^. The photocurrent external quantum yield is approximately 5.9 ± 0.3% within the low range of photo-intensities and decreases from 4.7 to 2.4% within the high range of photo-intensities.

As further evidence of the photo-intensities trend on a coated planar substrate, Fig. 2b shows the in situ gas-phase electrical conductivity sensitivity plots under an increasing flow ratio of CO_2_ and H_2_ to Argon (CO_2_ + H_2_)/Argon. Both CO_2_ and H_2_ gases are chemisorbed onto the In_2_O_3−*x*_(OH)_*y*_ nanorods coated surface and release free electrons to increase the measured current under an applied bias of 5 V. The slope of the current increase with the increasing concentration of reactant gases indicates the surface chemisorption sensitivity. The electrical conductivity increases below a threshold flow ratio of 0.005 under a low photo-intensity of 26 mW/cm^2^ and increases rapidly with increasing photo-intensities while the threshold value to saturation decreases to 0.023. This indicates that the population of surface chemisorbed gas states increases with increasing photo-intensities, but its maximum sensitivity is reached at approximately 104 mW/cm^2^ photo-intensity. The H_2_ component of the mixed atmosphere is more likely to be adsorbed than the CO_2_ component (Supplementary Figure 5) under thermal and photo-input, with the steepest increase at 150 °C and photo-illumination under 0.002 H_2_/Ar flow ratio.

The decrease in the activation energies from dark to photo-illumination relates to the glovebox protected XPS reactor-based measurements (Fig. 2c, d). For a quantitative estimate of the OH shoulder in the *O1s* spectrum, we define the ratio of OH shoulder area in relation to the *O1s* spectrum corresponding to the area integral between 531 and 534.5 eV in relation to the area for the spectral range from 528 to 534.5 eV. While a reactor temperature of 75 °C in the dark in a CO_2_ + H_2_ gas atmosphere does not result in significant changes to the *O1s* spectra in comparison with the as-prepared state, the increase in reactor temperature with a constant photo-intensity of 30.1 mW/cm^2^ shows that the OH shoulder area ratio under photo-thermal conditions is larger by 190–230% than that under thermal and dark conditions, with an associated indium binding energy increase of 0.15 ± 0.05 eV. There are two components within the changes in the OH shoulder: that of the peak intensity increase, and the expansion of the shoulder (Supplementary Figs. 6–8). The OH shoulder expansion is due to the development of H^+^OH sub-peak from H_2_ dissociation on OH sites, while increased peak intensity is mainly dominated by increased OH groups from H_2_ dissociation on oxide lattice sites.

To understand the link between photo-intensities and surface absorption of the gas reactants, we undertook XPS measurements of the coated waveguide and coated planar substrate post various photo-intensities and reactant atmospheres. The *O1s* spectra (Fig. 2e, g) show that with increasing incident photo-intensities the OH shoulder grows with intensities on both planar and waveguide substrates. With increasing photo-intensities there is also a small binding energy increase of 0.1–0.2 eV in the O_L_ lattice sub-peak and more significantly with ~0.3 eV in the OH shoulder peak, indicating that a greater fraction of H_2_ dissociation occurs on the OH group than on the oxygen lattice sites.

The quantitative growth of the OH shoulder with increasing photo-intensities was compared between the planar coated and waveguide coated geometries (Fig. 2h). The OH shoulder growth on the coated planar substrate is fairly linear at low to mid-photo-intensities but increases more slowly beyond 66 mW/cm^2^, which correlates approximately with the photo-current trend. For the coated rod waveguide, the OH shoulder area ratio of ~0.63 under an incident lamp photo-intensity of 82.6 mW/cm^2^ is equivalent to that of the coated planar substrate under an incident lamp photo-intensity of 60.2 mW/cm^2^. However, above an incident photo-intensity of ~95 mW/cm^2^, the OH shoulder ratio growth for the coated rod waveguide is more linear than the coated planar substrate. This is likely due to the effective lower photo-intensity exposed to the coating on the rod waveguide. Since the photon-excited-electron yield and photo-induced increase in OH species are more efficient at lower photo-intensities than higher photo-intensities, a greater mass of catalyst coated on a quartz rod waveguide can be illuminated with a photo-intensity that enables high photon to CO yield efficiencies. This results in an overall CO product rate improvement when the yield is normalized over the mass of the catalyst.

Overall, these results highlight the link between photo-generated charge carriers in the In_2_O_3−*x*_(OH)_*y*_ system and its effect on enhancing reactivity in the RWGS reaction. Indeed, the OH shoulder growth of the coated rod at the maximum photo-illumination is 294% greater than the OH growth associated with dark thermal conditions. In this context, photo-excited charge carriers significantly enhance the CO conversion rate, where photo-excited carrier hopping lifetime measurements being correlated with higher CO rates^21^. As well, reaction rate kinetic rate studies showed that photo-illumination increases the order of reaction of the H_2_ partial pressure from 0 to 1/3. Furthermore, transient absorption pump-probe spectroscopy showed that the fraction of the probe signal associated with long-lived excited states at the pump wavelength of 405 nm extends to 0.4–0.45 at a very low laser fluence of 0.20 mJ/cm^2^, but drops^27^ to 0.02 at a higher fluence of 1.33 mJ/cm^2^. This shows that long-lived charge states are more prevalent at low photo-intensities. The effect of low-intensity photo-illumination is to excite charge carriers on the surface that destabilizes charge neutrality and require charge carrier recombination through chemical adsorption of suitable reactant(s). The energetically shallow hole- capturing OH group and shallow electron-capturing unsaturated indium site generates a spatially separated charge pair that induces a polar electrical field across the SFLP site. Subsequently, by induction negatively charged In-H^−^ and positively charged H^+^OH groups are created through H_2_ heterolytic dissociation^28^. Because the presence of H^+^OH generates new mid-gap defect states within the bandgap, the subsequent adsorption of a CO_2_ molecule at the indium hydride and protonated hydroxyl group generates an intermediate that enables carrier recombination to take place therein. The intermediate state subsequently dissociates to CO and H_2_O. Hence, reinstatement of surface charge neutrality occurs through the adsorption of two reactants to form a new surface recombination center that acts as a separate recombination pathway other than the straightforward recombination pathway through the bulk.

### Optical scattering and waveguiding

**Fig. 3:**
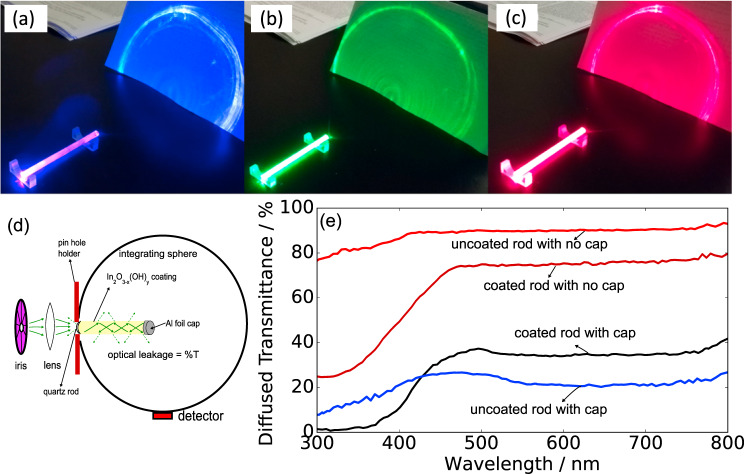
Spectral waveguiding. Waveguiding of blue (**a**), green (**b**), and red (**c**) laser illumination at an angle of incidence of 60°. **d** The integrating sphere measurement of the optical leakage through an In_2_O_3−*x*_(OH)_*y*_ coated cylindrical quartz rod waveguide, with **e** showing the optical leakage spectra through the sides (along the length) of a coated and uncoated cylindrical rod waveguide.

One unique aspect of our In_2_O_3−*x*_(OH)_*y*_ coated rod waveguide photoreactor is its waveguide behavior for non-absorptive and weakly absorptive wavelengths. Despite the porosity of the coating, red (732 nm) and green (535 nm) laser light at an incident angle of 60° can indeed propagate and internally reflect through the coated optical rod and transmit through to the other end (Fig. 3a–c). With blue laser illumination, the intensity decreases with the length of the rod, showing that the coating is partly absorbing blue light as it is internally reflected within the quartz rod. To distinguish the amount of light being transmitted through the rod or light reflected from the cylinder surface, we carried out an integrating sphere measurement of diffused transmittance (Fig. 3d) of coated/uncoated rods encapsulated and unencapsulated at the back end with reflective aluminum foil. The cap eliminates the transmittance of the spectrometer beam through a direct optical path and reflected optical paths, revealing the transmittance of the leakage through the sides of the cylindrical surface. Figure 3e shows that the catalyst coating itself has ~30–37% optical leakage for wavelengths greater than 500 nm. The direct transmittance through the end of the rod is 40–45% of the incident light. In contrast, the uncoated quartz rod is effective as a waveguide yielding nearly 90% transmittance, accounted for by the sum of the optical leakage through the sides and through the end of the rod. However, with the cap on the rod end the optical leakage of >500 nm light through the sides of the rod drops significantly to 18–27%. The optical leakage of the In_2_O_3−*x*_(OH)_*y*_ coated rod is thus comparable with that of the rod with no coating, hence the In_2_O_3−*x*_(OH)_*y*_ coating acts as a fairly effective waveguide in green and red wavelength regimes.

**Fig. 4:**
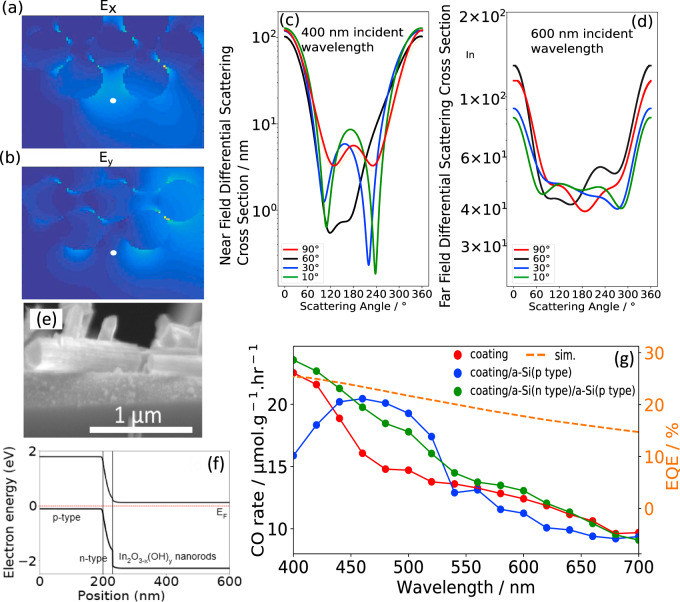
Nanorod optical scattering. **a**, **b** The optical Mie scattering simulations of an array of cylindrical rods with refractive index of indium oxide at 400 and 500 nm incident wavelength. The array of cylindrical rods of 60 nm diameters near the scatterer point is shown, as marked by the white dot, under a plane wave at an incident angle of 30° to the *x*-axis. The scattering intensity plots under the **a** *E*_x_ and *E*_y_ **b** components of the incident plane wave are shown, respectively. **c**, **d** The near field and far-field scattering cross-sections at a range of scattering angles under several incident angles from 90° to 10° from the positive *x*-axis plane with 400 and 600 nm wavelengths, respectively. **e** SEM cross-sectional images of nanorods on 200 nm p-type/40 nm n-type silicon layers. The white scale bar represents 1 µm length. **f** Calculated equilibrium band diagram of *p-*type a-Si/*n* type a-Si/In_2_O_3−*x*_(OH)_*y*_ system. **g** Photo-action spectroscopy of quartz rod coated with In_2_O_3−*x*_(OH)_*y*_ nanorods (red), nanorods on *p*-type amorphous silicon (blue), and nanorods on *n*-type/*p*-type amorphous silicon (green). The nanorods on *p*-type silicon have increased CO rates in the green regime, while nanorods on *n*-type Si/*p*-type Si have a broader increase in CO rates across the blue-green regime. The simulated EQE of an *n*-type Si/*p*-type Si/indium-oxide stack is shown in orange.

To determine if the nanorod geometry contributes optically to the propagation of light through the waveguide, the scattering behavior for the indium oxide hydroxide nanorod geometry was determined as a function of different incident angles, near the bandgap absorption wavelength of 400 nm for indium oxide hydroxide. Based on trends in optical scattering cross sections for an array of cylindrical rods (Fig. 4a) and given the refractive index of indium oxide, the rods exhibit high scattering at shallow incident angles of an incoming plane wave in the near and far-field (e.g., a near field scattering angle between 110° and 230° at an incident angle of 30°). For incident radiation at a wavelength of 600 nm and at 30° angle and other angles, the scattering cross-section width of 4–4.9 nm is fairly broad at all scattering angles, highlighting that scattering of these nanorods is fairly independent of incident angles at 600 nm wavelength. Since the nanorods have inherently greater scattering than spheres with the same diameter of 60 nm, by at least 1 order of magnitude in the far-field (Supplementary Fig. 11), hence the In_2_O_3−*x*_(OH)_*y*_ nanorods (Supplementary Fig. 12) scatter light effectively within the waveguide given that light leakage can be minimized by the reflective foil. Indeed, the optical path lengths within a rough film can be increased by a factor of $4n^2/{\mathrm{sin}}(2\theta )$, which is the well-known Lambertian limit, where *n* is the refractive index of the scattering film, and *θ* is the acceptance angle at which the light ray is incident onto the scattering film. The absorbance of the film is then $A\left( \lambda \right) = 1 - e^{ - 4n^2\alpha \left( \lambda \right)d}$ where $\alpha (\lambda )$ is the absorption coefficient dispersion (*α*(600 nm) = 277 cm^−1^) and d the film thickness, assuming no reflectance. Hence, at the wavelength of 600 nm, the absorbance of the catalytic film increases from 0.13 to a maximum of 0.76 for a thickness of 5 µm at the Lambertian limit.

We can exploit this visible wavelength scattering behavior in the quartz rod waveguide since In_2_O_3−*x*_(OH)_*y*_ has a weak and long optical absorption tail extending from the absorption edge of ~470–680 nm (Supplementary Fig. 13), which is attributed to Urbach tail states (associated with the aforementioned OH, O_vac_ defects) of lower energies within the bandgap. Further evidence of the extended band gap states was shown in the UPS measurements (Supplementary Figs. 6e and 7e). The valence band edge showed an absorption tail extending below 2 eV after photo-illumination in the reactant gas atmosphere. Hence, with a UV bandpass filter that absorbs wavelengths smaller than 410 nm, the CO rate for a coated planar substrate is 7.75 ± 0.32 µmol/g.cat.hr, but is higher by a factor of 4.6 (35.6 ± 0.21 µmol/g.cat.hr) for the encapsulated coated rod waveguide. With a yellow filter and a red filter that block out wavelengths smaller than 500 and 620 nm, respectively, the CO rates for the coated planar substrate are fairly small at 0.6–1.02 ± 0.2 µmol/g.cat.hr, but are higher by a factor of 8.7 (9.68 ± 0.52 µmol/g.cat.hr) and 8.1 (4.87 ± 0.34 µmol/g.cat.hr) for the coated rod waveguide, respectively, which together show that the optical absorption tail extends even beyond the yellow wavelength regime. These results show that the longer optical path lengths of the weakly absorptive wavelengths can be utilized to enhance CO production at sub-bandgap photon energies. In addition, future development of such rod waveguide photoreactors can include other photocatalysts absorbing in different wavelength regimes in order to create a “tandem” waveguide that allows the entire illumination spectrum to be harvested by In_2_O_3−*x*_(OH)_*y*_ and other photo-catalytic systems.

As an example of harvesting the visible wavelength regime by pairing In_2_O_3−*x*_(OH)_*y*_ with another material on a quartz rod, we coated the quartz rod waveguide with doped amorphous silicon via plasma-enhanced chemical vapor deposition. The In_2_O_3−*x*_(OH)_*y*_ nanorods are subsequently applied onto the silicon film and the waveguide encapsulated with Al foil. The CO rates for the 200 nm p+ doped silicon/In_2_O_3−*x*_(OH)_*y*_ system in the batch reactor are 4.31 ± 0.35 µmol/g.cat.hr and 12.54 ± 0.33 µmol/g.cat.hr under the red and yellow filters, respectively, thus showing a significant increase in conversion rates in the visible wavelength regime—specifically, by a factor of 1.28. Photo-action spectroscopy of CO yield as a function of wavelength shows that the *p*+Si/In_2_O_3−*x*_(OH)_*y*_ system produces significantly greater CO at wavelengths from 440 to 520 nm, specifically an increase of 19% over the coated quartz rod waveguide. The increase in the visible regime harvesting can be associated with the diode junction created between the p+ doped silicon and intrinsically n-type In_2_O_3−*x*_(OH)_*y*_ junction, which drives electrons from the silicon junction into the In_2_O_3−*x*_(OH)_*y*_ layer. Simulated EQE spectrum^29^ of the p–n junction showed a broad charge carrier generation across the visible spectrum, hence, in order to improve charge carrier separation (Fig. 4e, f), a thin n-doped silicon layer of 40 nm thickness was deposited on the *p*+ layer before coating In_2_O_3−*x*_(OH)_*y*_ nanorods. The *p*+Si/*n* Si/ In_2_O_3−*x*_(OH)_*y*_ showed a greater CO rate with increasing photo-intensities than the *p*+Si/ In_2_O_3-*x*_(OH)_*y*_ coated waveguide (Supplementary Figure 14). In addition, the resulting photo-action spectroscopy (Fig. 3g) of the *p*+Si/*n* Si/In_2_O_3−*x*_(OH)_*y*_ layer, showed increased production across the wavelength range of 400–560 nm, resulting in an overall UV to visible regime increase of 27% over the nanorod coated waveguide.

### Persistent photocatalysis

**Fig. 5:**
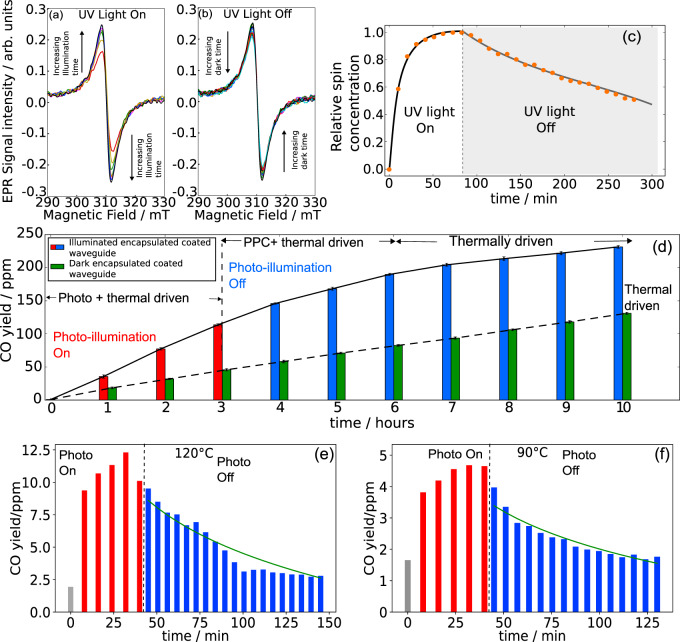
Persistent photo-catalysis. EPR spectra of In_2_O_3−*x*_(OH)_*y*_ nanorods under UV illumination (**a**) and post illumination (**b**) with time intervals of 5 min. **c** The change in spin concentrations normalized over the maximum spin concentration. **d** The CO yield with time for an aluminum foil encapsulated In_2_O_3−*x*_(OH)_*y*_ coated waveguide in batch reactor configuration, under photo-illumination for 3 h and post photo-illumination, under 200 °C reactor temperature. Persistent photoconductivity (PPC) enables a comparable yield to increase immediately after photo-illumination, which is greater than that of the dark condition under the same reactor temperature. **e**, **f** The CO yield decay after post-illumination in flow reactor configuration, at reactor temperatures of **e** 120 °C and **f** 90 °C. The regression fitted exponential decay trends are shown in green lines.

An intrinsic material advantage of using In_2_O_3−*x*_(OH)_*y*_ nanorod coatings is its persistent photo-conductivity effect^30,31^, as shown earlier in Fig. 2a—inset, which showed that photo-current values saturate within 2 h of photo-illumination but slowly decays to its original dark current value in 7 h or more. Indeed, electron paramagnetic resonance (EPR) spectra (Fig. 5a) of the nanorods under 405 nm laser illumination exhibit a rapid increase in signal intensity within 15 min, followed by a gradual decrease in signal intensity post illumination (Fig. 5b, c). The EPR signal of indium oxide is commonly associated with singly ionized oxygen vacancy defects^32,33^, which implies the photo-ionization of neutral oxygen vacancies, with trapped electrons that are slowly released post-illumination.

Figure 5d shows the results of regular and persistent CO production rate during and post photo-illumination respectively, where the time-accumulated CO product yield of the aluminum foil encapsulated In_2_O_3−*x*_(OH)_*y*_ coated rod waveguide increases from 0.7 to 114.6 ppm within 3 h of 107 mW/cm^2^ photo-illumination at 200 °C. The CO production average rate is ~38 ppm/h, yet increases at a comparable averaged rate of 23.4 ppm/h after turning off photo-illumination for a period of 2 h. Subsequently, the rate increase is smaller and linear at 11.9 ppm/h. By comparison, the thermal only reactor condition of 200 °C under dark showed a linear averaged CO product yield increase of 14.1 ppm/h, which is in line with the yield increase rate post 3 h after turning off photo-illumination. The averaged CO rate is thus enhanced by a factor of 1.62 over the averaged thermal rate within the persistent period. In the flow reactor configuration, the coated waveguide rod exhibits a CO decay period of 35–45 min at a reactor temperature of 120 °C (Fig. 5e), and 50–55 min at a reactor temperature of 90 °C (Fig. 5f). The averaged CO yield over the decay period is greater than the average thermal only yield by a factor of 1.44 and 1.57 for 90 and 120 °C, respectively. Since the photo-thermal associated temperature of In_2_O_3−*x*_(OH)_*y*_ was determined to be less than 20 °C under 0.8 sun illumination, it is unlikely that the persistent photo-catalysis is due to residual thermal effects after turning off photo-illumination.

**Fig. 6:**
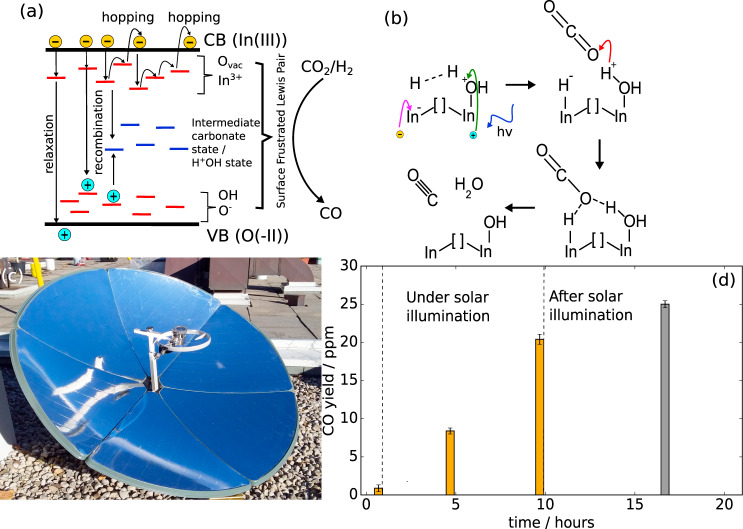
Outfield test. **a** An illustration of a band diagram where the various defect states populate the bandgap as shallow and deep energy traps. A photo-excited electron carrier can decay through recombination or relaxation, or be trapped in shallow states and undergo hopping which can lead to re-emission into the conduction band edge. Each hopping step into a neighboring trap requires a time interval, resulting in a persistent population of excited carriers after photo-illumination is off. **b** The reaction mechanism of an SFLP site consisting of an indium, oxygen vacancy, and hydroxyl group site. Photo-excited charge carriers within the bulk are injected into surface OH groups, followed by heterolytic splitting of an incident H_2_ molecule into a proton and hydride. Subsequent formation of a carbonate dissociates oxygen from the carbon dioxide to form CO and H_2_O. **c** The rooftop solar concentrator and reactor attached to it. **d** The CO yield of the encapsulated coated rod waveguide after 9 h of solar illumination and post-illumination.

Assuming a time-dependent exponential behavior, the rate of decay has an exponential decay constant of 5.10 and 1.73 min, respectively. However, the fit is not accurate with the experimental decay profile, indicating a sublinear decay trend which is indicative of a continuum of band defects with a range of trap energies^34^, since a single trap energy level results in an exponential decay trend in photocurrent after turning off photo-illumination. With a continuum of trap states that are distributed close to each other in energy and spatially proximal, successive hopping of trapped electrons can re-emit electrons back into the conduction band, resulting in a persistent population of excited charge carriers after photo-illumination (Fig. 6a). Due to the population of excited charge carriers, charge injection into a surface OH group, as well as the coordinately unsaturated indium site (Supplementary Fig. 17), can induce heterolytic dissociation of the H_2_ molecule (as described earlier) which subsequently reacts with CO_2_ through the H^+^OH indium terminated and H^−^ indium terminated species to form CO and H_2_O products of the RWGS reaction (Fig. 6b).

Overall, the In_2_O_3−*x*_(OH)_*y*_ waveguide photoreactor is suitable as an integrated solar concentrator batch reactor (Fig. 6c) with no external heating, where the CO rate can remain relatively high even after sunset or during cloudy periods in the day. As an example, we operated a solar batch concentrator (without solar tracking) during daylight hours and then continued to monitor the CO yield in the night post-sunset. As shown in Fig. 6d, through the course of the night the CO yield increased by 19% (24.05 ± 0.22 ppm)—over and above that measured at the conclusion of the sunlit hours (some 9 h of solar illumination yielding 20.45 ± 0.43 ppm; the rate of 8.89 ± 0.25 µmol/g.cat.hr).

The ability to slowly release charge carriers from trap defect states for further chemical reactions occurs for an all-organic poly-heptazine imide^35^. Herein it is established that an all-inorganic defect-laden In_2_O_3−*x*_(OH)_*y*_ photocatalyst can prolong solar fuel generation and overcome the “duck curve” issue associated with on-demand energy utilization during low solar illumination in the evenings, a step-towards solving the intermittency problem for future solar fuels farms.

## Discussion

In this study, we show that the principle of light concentration yields diminishing returns on product yield, whereas applying a principle of light maximization enables significantly higher photon-to-yield efficiencies and higher catalysis rates. High efficiencies and high rates are not possible under straightforward light concentration due to the volumetric generation of charge carriers over-saturating the surface density of catalytic active sites, as well as charge carrier recombination rates increasing with increasing photo-intensities. When applied on a simple cylindrical quartz rod waveguide, defect laden In_2_O_3−*x*_(OH)_*y*_ serves as a suitable photocatalyst for the RWGS reaction at mild reactor temperature. In particular, we show markedly improved performance owing to several factors pertaining to optical-photocatalytic synergy. The photo-current and XPS derived OH species spectra showed that on the coated waveguide, the photo-generated surface electron concentration efficiently maximizes the activation of surface catalytic sites. Further, the optimization of the nanorod geometries enables wide-angle scattering for efficient light propagation through the waveguide, which enables a large area of catalyst material to be illuminated with optimal light intensities. The propagation and internal reflection of green and red wavelengths enable sub-gap trap states to be occupied continuously, which can enable conduction band electrons and valence band holes to recombine through the surface catalytic pathway. The presence of sub-gap trap states can be further exploited through the phenomenon of persistent photo-conductivity.

Future development of photocatalyst-coated waveguides should take advantage of the ability to maximize photocatalytic performance over a large surface to volume ratio. In addition, tandem photocatalytic layers can be laterally coated so different sections of the waveguide can utilize various regimes of the wavelength spectrum. To maximize catalyst loading and catalyst-gas contact, several coated tandem waveguides integrated into a compact photo-reactor configuration (e.g., shell and tube), in which space minimization between waveguides, can potentially allow any transmitted refracted light from one coated layer to be used by another coated layer. As presented in this work, compact photoreactor configurations integrated with solar concentrators, result in both higher quantum efficiencies and spectral utilization. Moreover, due to the improved CO production rate from waveguide support, the intrinsic property of persistent photocatalysis in In_2_O_3−*x*_(OH)_*y*_ can be exploited to enable solar fuel production after sunset.

## Methods

### Reactor measurements

For flow reactor measurements, a flow rate of 5 sccm H_2_ and 1 sccm CO_2_ was used at 14.1 psi with custom-built flow controllers. The flow reactor comprises a flange with a glass viewport, with a side gas connection on the flange for the input flow, and a tube connected to the bottom of the flange enables the aluminum foil encapsulated coated waveguide rod to be squeezed into the tube, such that the front of the waveguide rod is exposed to the glass viewport only. A thermocouple at the back end of the tube would be in contact with the surface of the coating on the back end of the waveguide rod. For persistent CO yield measurements, the reactor would be under high temperature and gas flow for at least 3–4 h so that thermal equilibrium is established before turning on photo-illumination.

For batch reactor measurements, the reactor would be vacuum pumped to a pressure of 2 × 10^−2^ mbar under the set reactor temperature and under the lowest intensity photo-illumination, followed by H_2_ introduction to a pressure of 26–28 psi, for a period of 5 min, before the introduction of CO_2_ to a pressure of 48–52 psi. The time at which the CO_2_ is introduced is considered the start of the reaction cycle. The reactor would be under temperature and photo-illumination for an hour before GC measurement. At least three subsequent data points would be collected at every hour, in order to generate the uncertainty range. The photo-intensity would be increased with an interval step of 15–20 mW/cm^2^, and the reactor reset to vacuum state for the next set of measurements as discussed above. The aluminum foil encapsulated coated waveguide rod would be set in the center of the reactor by affixing the rod partly through a center hole in the cap of the reactor backend.

### Electrical measurements

For the electrical conductivity-gas sensitivity measurement, a modified cryostat was used to hold the sample with 4 probe needles on a set of square gold electrodes 0.5 mm distance apart in a square configuration. For each temperature and photo-intensity measurement, a separate set of 0.5 mg powder would be used. The cryostat would be pumped to ~1 × 10^−2^ mbar before introducing a 1000 sccm flow of Ar and 1 sccm flow of H_2_ and 1 sccm flow of CO_2_. The argon flow would be varied using a custom potentiostat to reduce the flow of Argon while maintaining the same flow rates for H_2_ and CO_2_. Two hours of photo-illumination at the set temperature would take place under vacuum, before carrying out the electrical conductivity-gas sensitivity measurements.

### Silicon deposition

PECVD a-Si:H Deposition parameters before coating with indium oxide nanorods: The quartz rods were cleaned using Acetone, Methanol, and DI Water. The quartz rods were immersed in each solvent in an ultrasonic cleaner for 15 min, then were additionally rinsed with DI water and blown dry with nitrogen. For the deposition of a-Si:H, an RF PECVD chamber on an MVS Systems cluster tool was used. For each doped layer, there were two depositions in total, one on the cylindrical surface directly exposed to the chamber, followed by a rotation to expose the other half-side of the cylindrical surface. The sample deposition parameters are as follows. The p-doped a-Si:H and n-doped a-Si:H, respectively, has electrode spacing 34 mm each, temperature 160 and 180 °C, SiH_4_ flow rate of 8 sccm each, the pressure of 0.175 Torr each, RF power of 10 W and 2 W, deposition time of 20 min and 25 min. The flow rate of 1.98% B_2_H_6_ in H_2_ flow is 20 sccm for p-doped and the flow rate of 1.88% PH_3_ in H_2_ flow is 20 sccm for n-doped a-Si:H. For characterization of film conductivity parameters, *n*-type and *p*-type films were deposited on glass substrates. These substrates were cleaned in the same manner as the glass rods. The deposition parameters also remained the same, except the *p*-type film was deposited for 30 min.

### Silicon electronic parameters

To measure the a-Si:H film conductivity, transmission line measurements were performed, with pad sizes of 2 mm (width) × 2.5 mm (Length) with gaps varying from 0.2 to 0.6 mm in steps of 0.1 mm. For the *p*-type film, 300 nm thick aluminum contacts were deposited using RF sputtering (300 W, 5.0 mTorr, Ar flow rate of 20 sccm). For the *n*-type film, silver contacts were deposited using the same method (300 W, 5.0 mTorr, Ar flow rate of 20 sccm). Doping concentration and mobility were not measured. Instead, the doping percentage was estimated using the fit line from a previous study on PECVD doping efficiency. We also expect the density of amorphous silicon to be in the range of 0.7–0.9 of the density of crystalline silicon, so 0.8 was taken as an average value. The atomic density of silicon is 5.0 × 10^22^ cm^−3^. Multiplying these gives us the expected doping concentrations. The mobility was estimated with *σ* = *qµn*. The n-type and p-type have carrier concentrations and mobilities respectively as 3.9 × 10^18^ cm^−3^ and 1.5 × 10^−3^ cm^2^ V^−1^ s^−1^, 8.5 × 10^18^ cm^−3,^ and 5.3 × 10^−5^ cm^2^ V^−1^ s^−1^. The estimated mobilities are comparable to those reported in the literature. The mobility of holes in amorphous silicon is well established as being orders of magnitude lower than that of electrons.

### Simulated quantum efficiency

In order to simulate the quantum efficiency spectrum of a back-illuminated *p*-type/*n*-type/Indium oxide stack, we employed Solcore, which is a comprehensive solar cell simulation package, with the stack of *p*-type amorphous silicon as the emitter, *n*-type amorphous silicon as the base, and Indium oxide stack as the window. The dopant concentration of the 30 nm thick *n*-type is 3.9 × 10^18^ cm^−3^ and 8.5 × 10^18^ cm^−3^ for the 200 nm thick *p*-type layer. In order to determine the equilibrium bandgap of the p/n/In_2_O_3_ junction, a 1D Poisson equation solution was implemented in Python, with the amorphous silicon dopant concentration of 4 × 10^18^ cm^−3^ (*n*-type) and 9 × 10^18^ cm^−3^ (*p*-type), with In_2_O_3_ *n*-type dopant averaged concentration of 1 × 10^16^ cm^−3^ as determined from Hall mobility measurements in prior work (ranges from 5.2 × 10^15^ cm^−3^ to 1.4 × 10^16^ cm^−3^ over the temperature range from ambient to 150 °C under photo-illumination).

### Experimental quantum efficiency

The amount of photon energy absorbed by the In_2_O_3−*x*_(OH)_*y*_ coating is approximated by $N_{aph} = \left( {E\left( \lambda \right) \times A_{\rm{rod}}} \right)/U_\lambda$; where *E*(*λ*) represents the fractional difference in light intensity transmitted by the coated and uncoated rod and can be determined by integrating the arc lamp spectrum with the transmittance spectra of the coated or uncoated rod shown in Supplementary Fig. 15 and Fig. 3e, *A*_rod_ is the surface area of the rod excluding the ends, and $U_\lambda = {\int}_{{\mathbf{\lambda }}_2}^{{\mathbf{\lambda }}_1} {(h.c \times N_A)/\lambda d\lambda }$ is the photon energy (J/mol photon) summed over the UV–visible regime over the xenon-arc lamp, where *h* is the Planck constant (6.626 × 10^−34^ J s), *c* the speed of light (3 × 10^8^ m/s) and *N*_*A*_ is Avogadro’s number (6.022 × 10^23^ mol^−1^).

### EPR measurement

The EPR system was a custom instrument with *ν* = 9342.4 MHz. By fitting the EPR signal with Easyspin, the concentration of spin states (*G* factor of 2.152 with a non-Gaussian linewidth of 5.3) increased to 160% of the dark spin concentration after an hour of illumination and decays sub-linearly after turning off photo-illumination. Indeed, the concentration of spin states remains high at 121% of the dark concentration after 200 min, thus indicating a slow release of photo-ionized oxygen vacancies to neutral oxygen vacancies. Extrapolating the decay trend shows that it will take 410 min after turning off photo-illumination to reach the original dark concentration of spin states.

## Supplementary information

Supplementary Information

## Data Availability

All data were available upon reasonable request.

## Code availability

All code available upon reasonable request.
