# Supplementary material for: Waveguide photoreactor enhances solar fuels photon utilization towards maximal optoelectronic – photocatalytic synergy
Source: Nat Commun. 2021 Jan 15;12:402. doi: 10.1038/s41467-020-20613-2 (PMC7810999; doi:10.1038/s41467-020-20613-2)
Supplement: Supplementary file 1 — Supplementary Information [file 41467_2020_20613_MOESM1_ESM.pdf]

SUPPLEMENTARY INFORMATION FOR:

Waveguide Photoreactor Enhances Solar Fuels Photon Utilization Efficiency: Towards Maximal Optical – Photo-catalytic Synergy, by Joel Y.Y Loh, Abhinav Mohan, Andrew G. Flood, Geoffery A. Ozin*, Nazir P. Kherani*

Type of optical support	Property and Results	Improvement Factor	Wider Energy Implications
Planar	Green-red light: 1.0 µmol/g.cat.hr ;<br>Red light: 0.6 µmol/g.cat.hr ;	-	Visible light harvesting with a predominantly UV absorbing photocatalyst without doping problems.
Waveguide	Green-red light: 9.7 µmol/g.cat.hr ;<br>Red light: 4.9 µmol/g.cat.hr ;	× 9<br>× 8	
Planar	High photo-intensity: 20 µmol/g.cat.hr ;	-	Higher photon to catalysis efficiency at high photon intensities. Suitable for solar concentrators.
Waveguide	High photo-intensity: 49 µmol/g.cat.hr ;	× 2.5	
Planar	Intensity threshold before lower chemical sensitivity: 66 mW/cm^2.	-	Higher chemical sensitivity to reactants at high photon intensities. Improved understanding of light-intensity-chemistry.
Waveguide	Intensity threshold before lower chemical sensitivity: 107 mW/cm^2.	× 1.6	
Nanoparticle coating	Optical scattering, green light transmission: 0.1 nm.	-	Significantly greater light transmittance in waveguide photo-reactor. Compact optical reactors.
Nanorod coating	Optical scattering, green light transmission: 40 nm.	× 400	
Single coat waveguide	Integrated blue-green photo-action: 66 µmol/g.cat.hr.		Tandem layers for broadband spectrum harvesting. Potential for multi-layer catalyst-light-absorbers.
Tandem waveguide	Integrated blue-green photo-action: 101 µmol/g.cat.hr.	× 1.5	
Planar	Persistent Photo-catalysis period: 25 min.	-	Overcoming intermittent solar light from clouds and darkness, making solar fuels in the dark.
Waveguide	Persistent Photo-catalysis period: 50 min.	× 2.0	

Supplementary Table 1. Report card encompassing the improvement factors and multiple advantages a waveguide photo-reactor will have over a planar photo-reactor configuration.

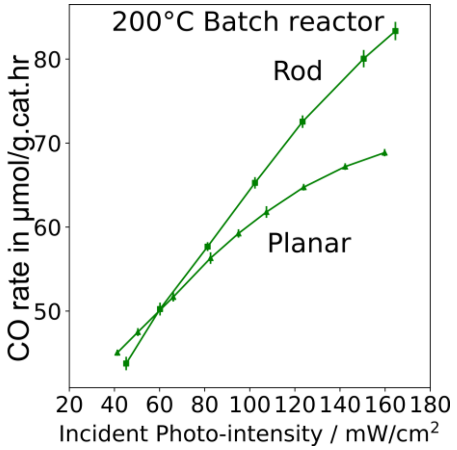

Supplementary Figure 1. CO rate at 200C in batch reactor configuration for coated rod waveguide and coated planar substrate. The maximum CO rate is 83.3µmol/g.cat.hr, representing an increase of 22% over the coated planar substrate.

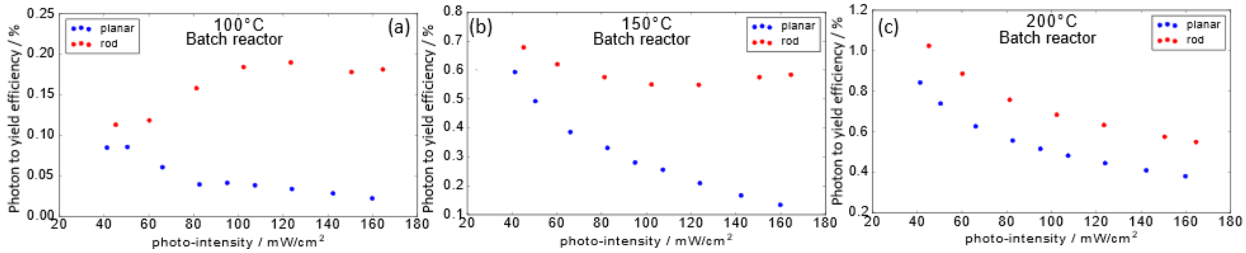

Supplementary Figure 2. The CO photon to yield efficiency of the coated planar and coated rod geometries, with increasing photo-intensities and at various batch reactor temperatures. The CO quantum efficiencies plateau at high photo-intensities at 150°C for the coated rod but continue to decrease for the coated planar geometry.

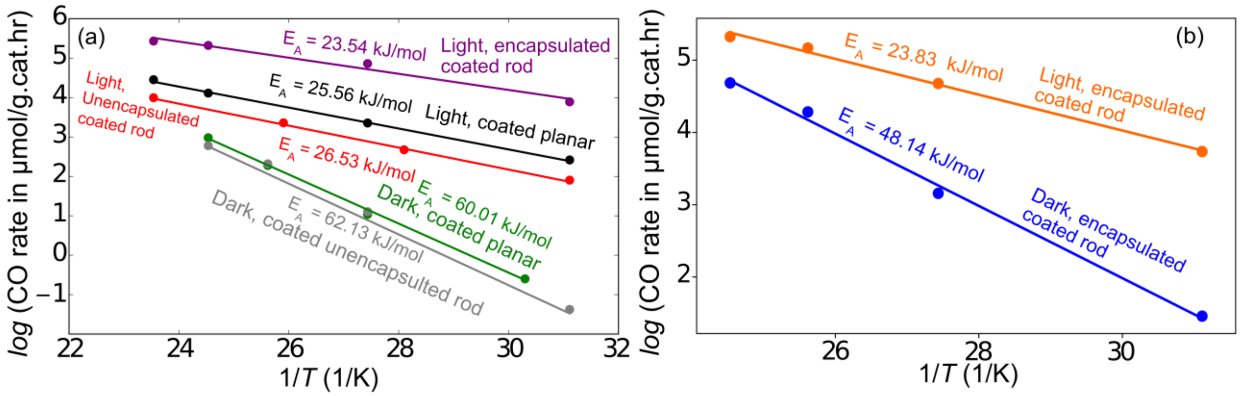

Supplementary Figure 3. Shows that the flow reactor associated activation energy (b) in the dark is slightly lower than the batch reactor configuration (a), while the photo-illumination associated activation energy is similar to that of the batch reactor.

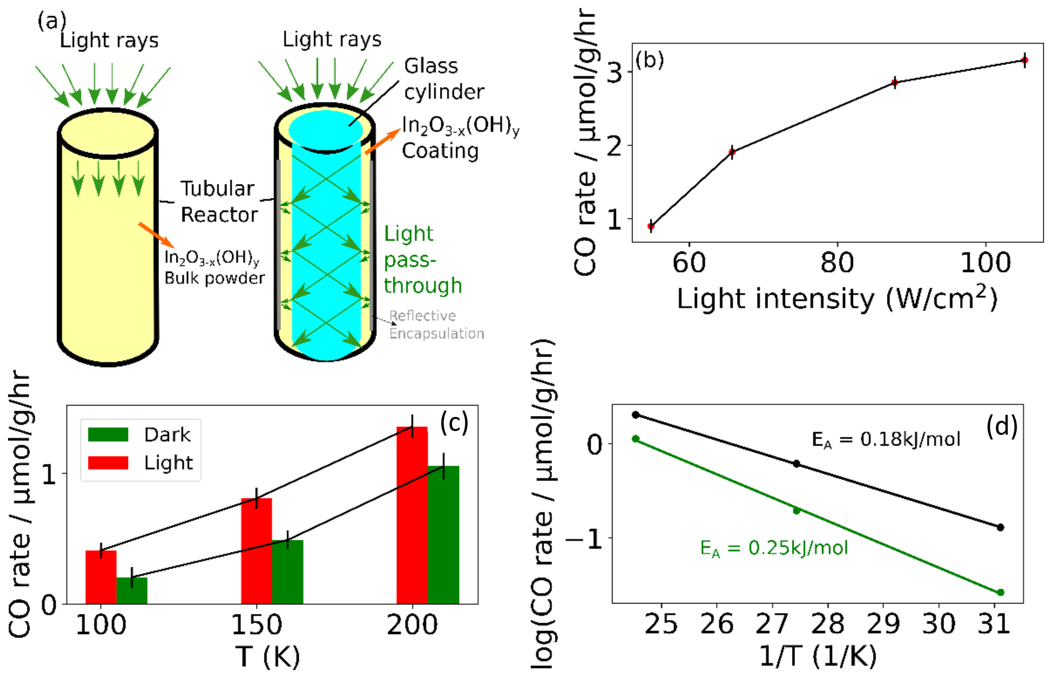

Supplementary Figure 4. A) Schematic showing how light rays are not able to penetrate deeply through a packed powder tube in a tubular flow reactor, whereas light guiding distributes light throughout the coating. (b) The CO rate at increasing light intensities through a packed bulk powder. (c) The CO rate at increasing temperatures. (d) The calculated activation energies at dark (green) and light (black).

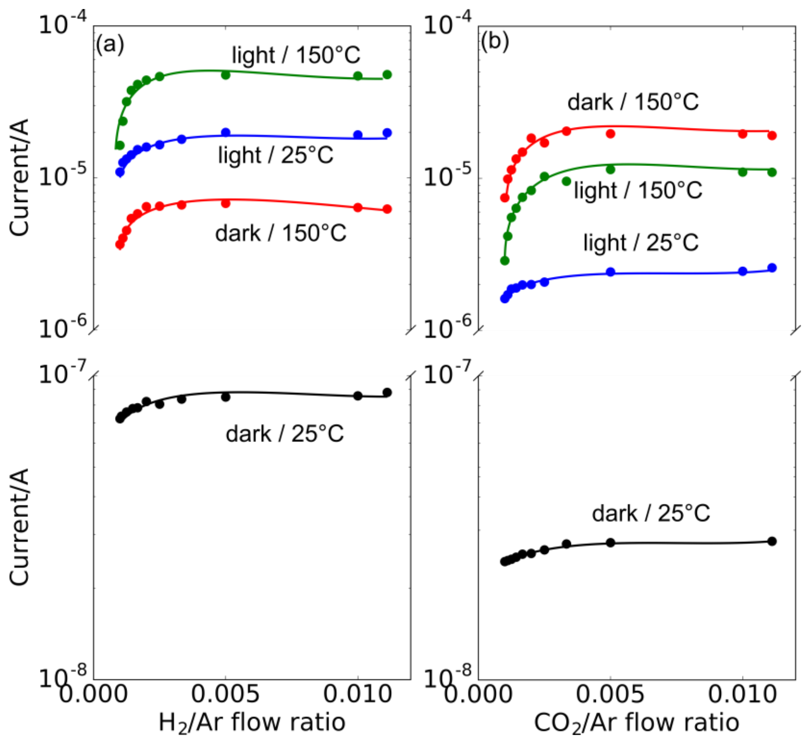

Supplementary Figure 5. (a) The electrical current measurements (log scale) at an applied bias of 5V for $In_2O_{3-x}(OH)_y$ nanorods with increasing concentration of H_2 (a) or CO_2 (b) in an argon flow, under the conditions of dark/ambient temperature (black), dark/150°C (red), photo-illuminated 25°C (blue), and photo-illuminated 150°C (green). The photo-illumination intensity was approximately 105 mW/cm^2. For H_2/Ar flow ratios, the greatest H_2 gas adsorption happens under photo-illumination and 150°C which is the reactor condition. However, for CO_2/Ar flow ratios, photoillumination reduces the surface adsorption of CO_2 in comparison with thermal condition, and that CO_2 adsorption under dark 150°C is greater than that of photo-illuminated 150°C. This indicates that CO_2 formation is favorable at high temperatures but photo-illumination desorbs carbonates.

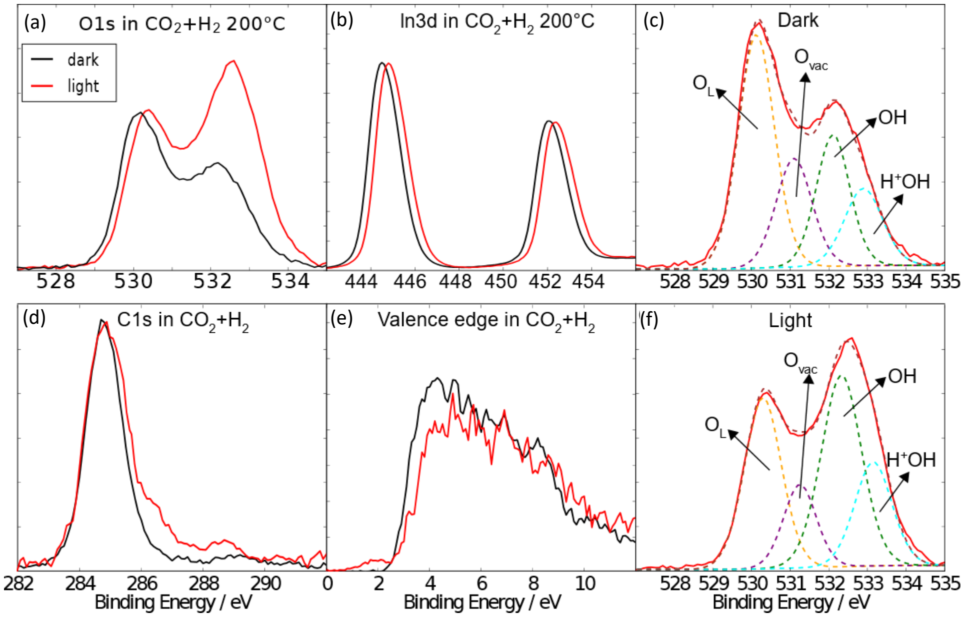

Supplementary Figure 6. The O1s (a), In3d (b), C1s (d) and valence band edge (e) spectra of $In_2O_{3-x}(OH)_y$ nanorods after 200°C in dark (black) and photo-illuminated (red) conditions. The deconvoluted spectra of dark and light conditions are shown in (c) and (f) respectively. To be noted, the valence band edge shows tail states after photo-illumination at 200°C, and the ratio of OH group is 0.207 and H^+OH is 0.095 for the dark condition, the ratios for the light conditions are 0.435 and 0.143, respectively.

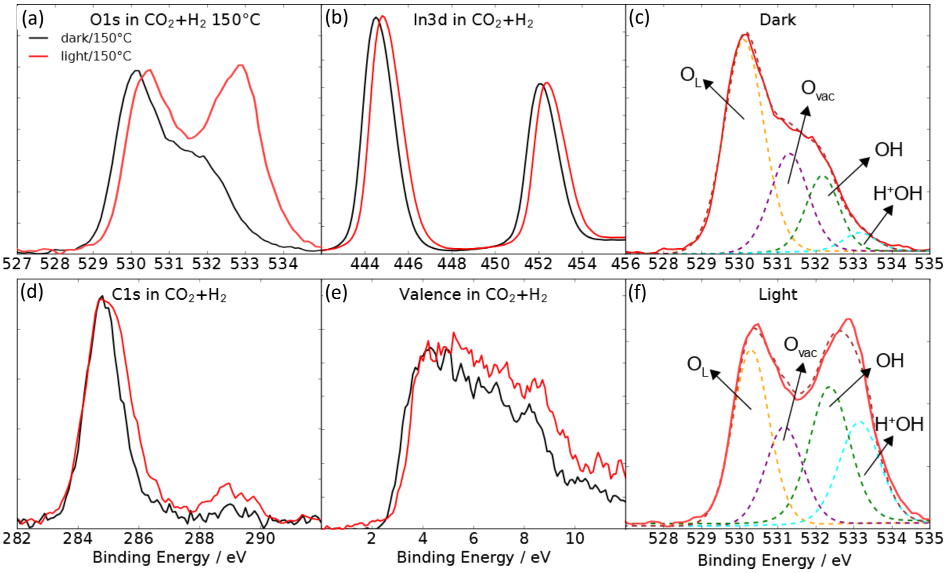

Supplementary Figure 7. The O1s (a), In3d (b), C1s (d) and valence band edge (e) spectra of $In_2O_{3-x}(OH)_y$ nanorods after 150°C in dark (black) and photo-illuminated (red) conditions. The deconvoluted spectra of dark and light conditions are shown in (c) and (f), respectively. The OH and HOH ratios of dark condition are 0.19 and 0.05; and light condition are 0.30 and 0.19.

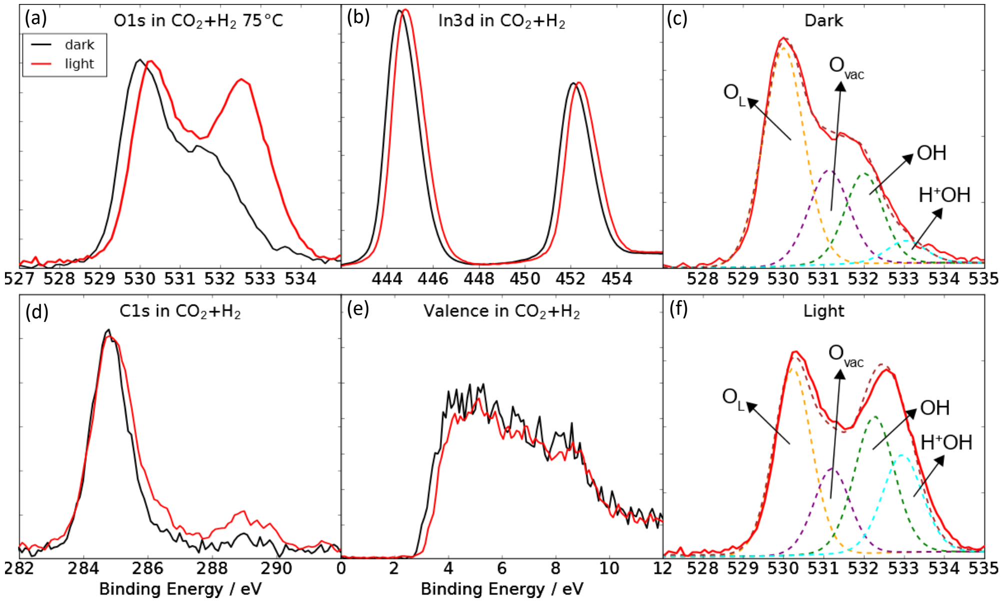

Supplementary Figure 8. The O1s (a), In3d (b), C1s (d) and valence band edge (e) spectra of $In_2O_{3-x}(OH)_y$ nanorods after 75°C in dark (black) and photo-illuminated (red) conditions. The deconvoluted spectra of dark and light conditions are shown in (c) and (f), respectively.

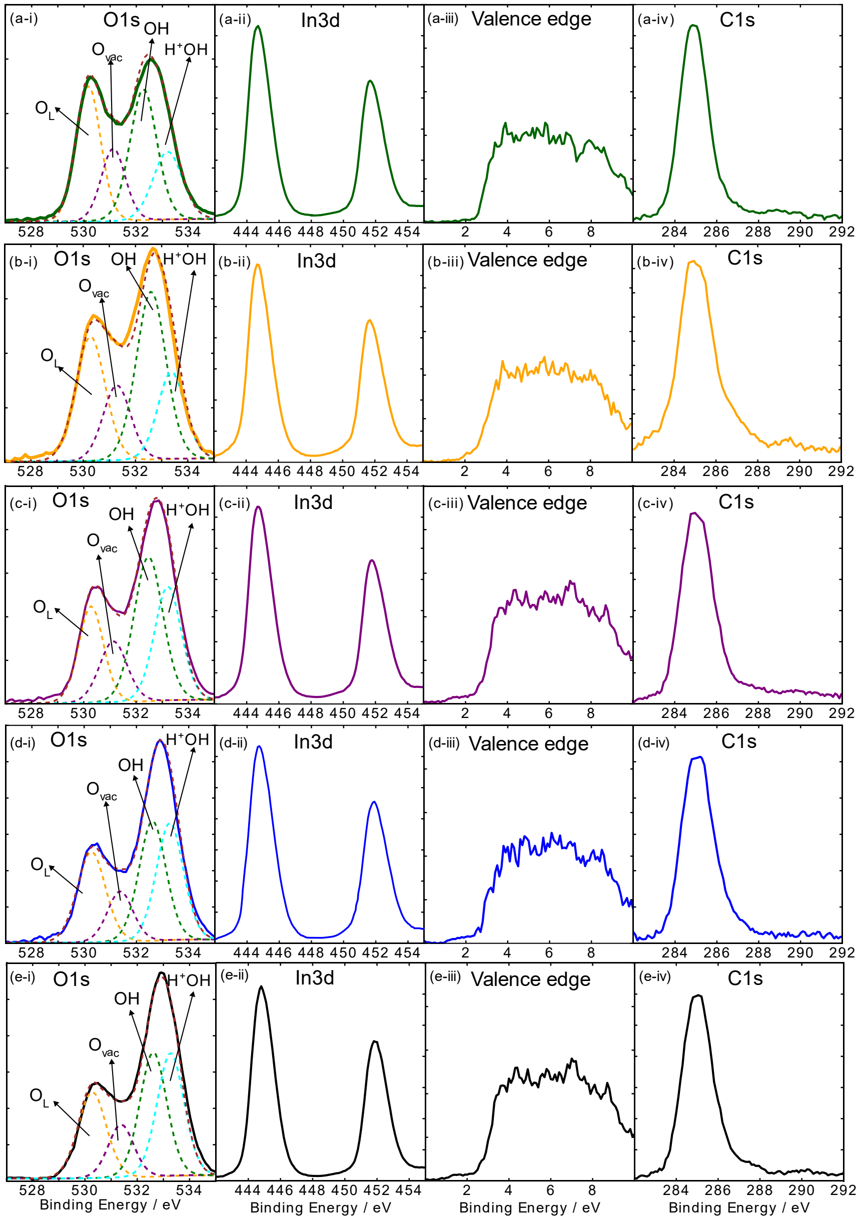

Supplementary Figure 9. Glovebox protected XPS measurements on $In_2O_{3-x}(OH)_y$ coatings on planar glass substrate with increasing photo-intensities (from (a) to (e)) at 200°C. (i-iv) represent the spectra for O1s, In3d, valence band edge and C1s respectively. With increasing photo-intensities, both the OH and H^+OH subpeaks increase in amplitude, with the H^+OH subpeak increasing in amplitude more-so than OH subpeak until its sub-peak area is similar to that of the OH sub-peak. The valence band edge also develops a low energy tail beyond 60.2 W/cm^2 photo-intensity. Since the C1s peak does not change significantly with increasing photo-intensities, the energy tail is likely associated with OH and H^+OH species.

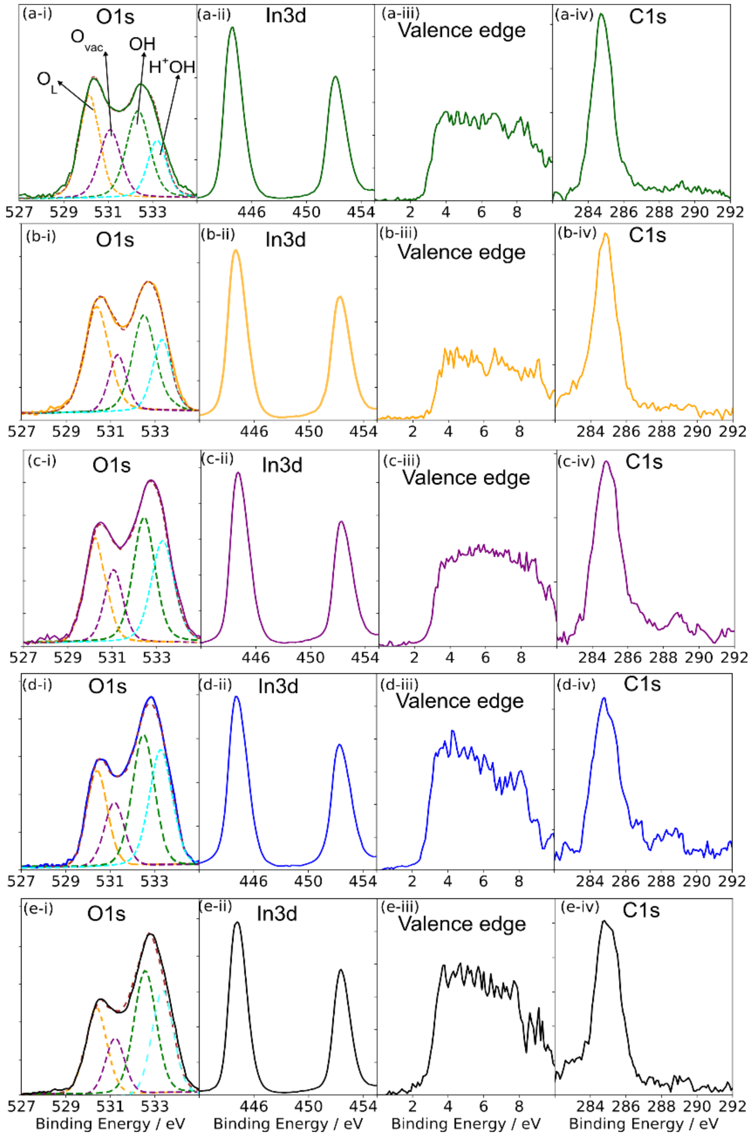

Supplementary Figure 10. Glovebox protected XPS measurements on $In_2O_{3-x}(OH)_y$ coatings on quartz glass rod waveguide substrate with increasing photo-intensities (from (a) to (e)) at 200°C. The width of the OH shoulder is wider than that of the planar substrate results, which leads to a greater OH shoulder area ratio at high photo-intensities compared to the coated planar substrate.

Supplementary Note 1

To determine optical scattering of a simple array of nanorods, we used the Matlab module MatScat. For the Mie Scattering calculation, the refractive index at 400nm and 600nm are assumed to be 1.7 and 1.6, respectively. The extinction coefficients are assumed to be 0.1 and 0.0013, respectively. The scattering cross sectional widths for near field and far field for the array of nanorods is shown in **Supplementary Figure 11**.

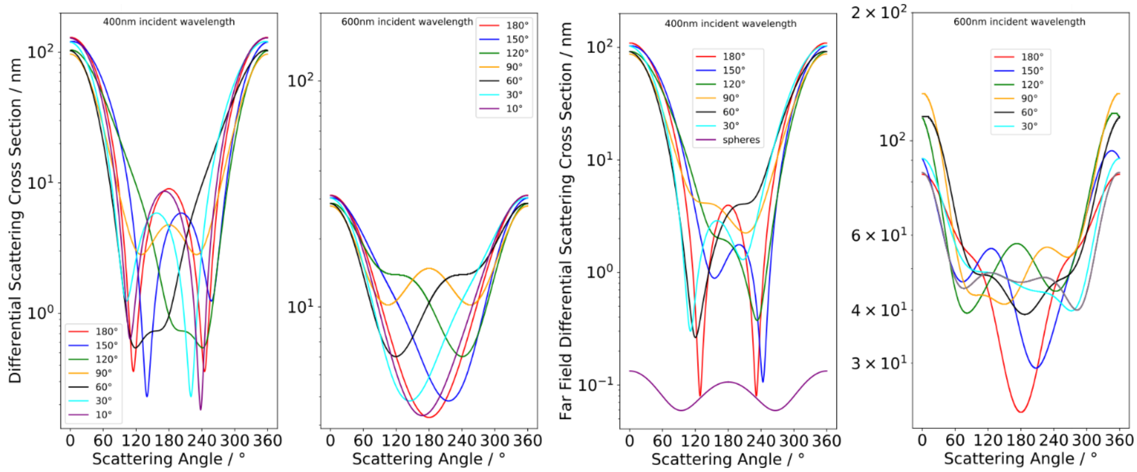

Supplementary Figure 11. Optical differential near-field (left) and far-field (right) optical cross section widths of the nanorod array under various incident angles from the positive x-axis.

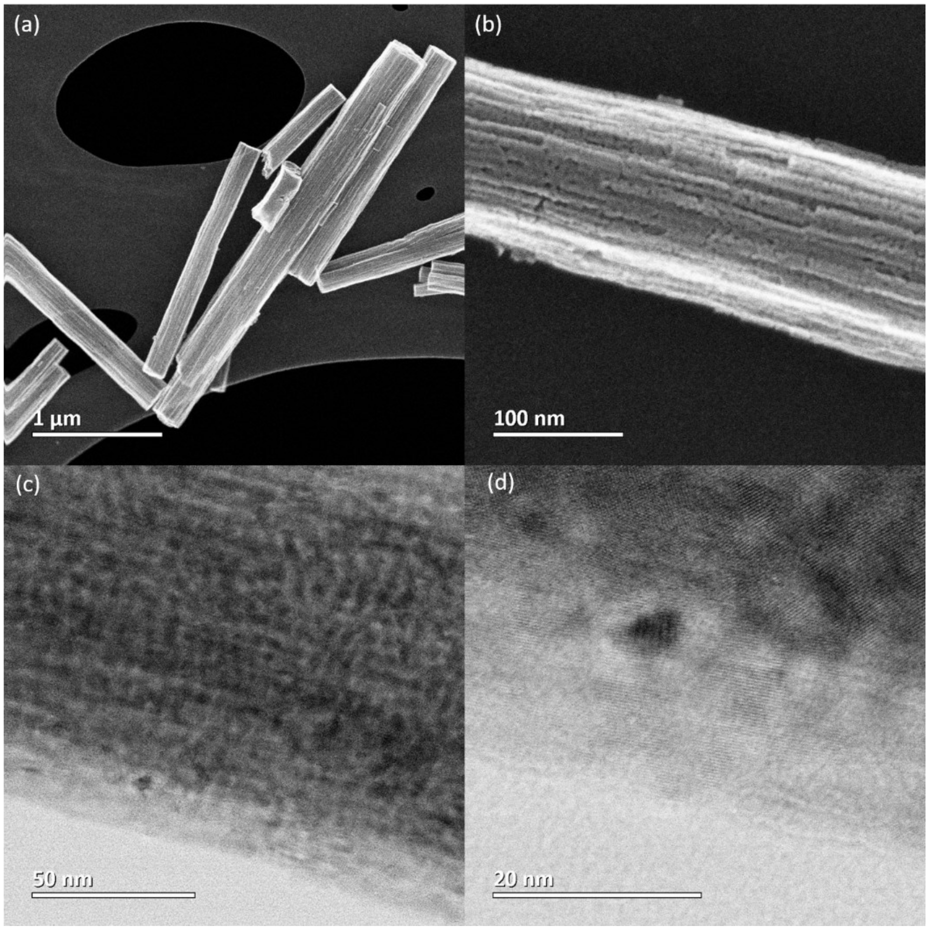

Supplementary Figure 12. TEM images of the nanorods. Magnified images in (b) show that one nanorod consist of multiple layers of nanoparticles bundled together as a nanorod superstructure. (c) and (d) show the atomic lattice.

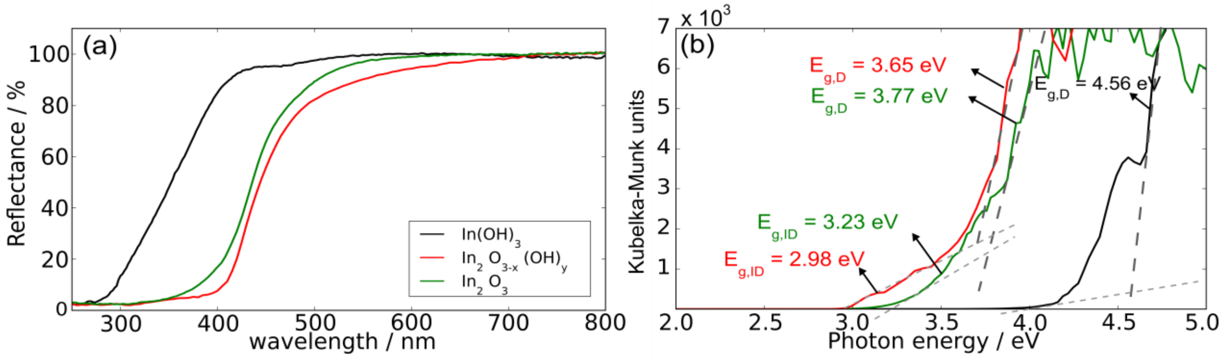

Supplementary Figure 13. The optical diffused reflectance spectroscopy (a) and Kulbeka-Munk determination (b) of the indirect and direct band gap values for the Indium hydroxide precursor $In(OH)_3$ (in black), sample of interest $In_2O_{3-x}(OH)_y$ nanorods (in red) and commercial powders (American Elements^{TM}) indium oxide In_2O_3 (in green). It is clearly seen that there is a small (15% absorption) but extended tail in the visible from 470nm to 700nm for the $In_2O_{3-x}(OH)_y$ nanorods. The KM plots show that the edge of the direct and indirect optical band gap of $In_2O_{3-x}(OH)_y$ begins at 3.65eV and 2.98eV respectively, while the commercial powder presents both a larger indirect and direct optical band gap.

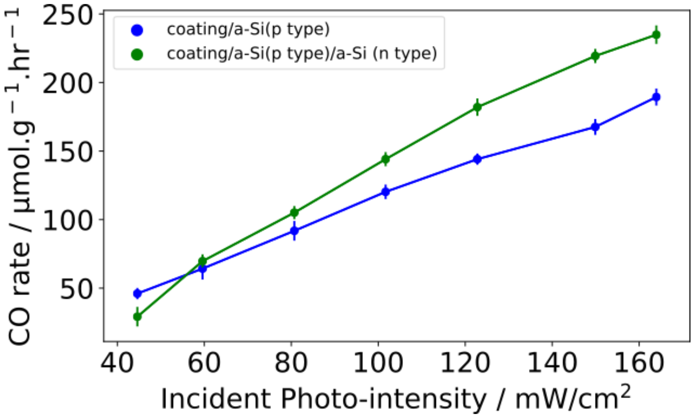

Supplementary Figure 14. The CO rate under various photo-intensities for the nanorod coatings on p-type and p-type/n-type amorphous silicon deposited layers on quartz rod waveguides.

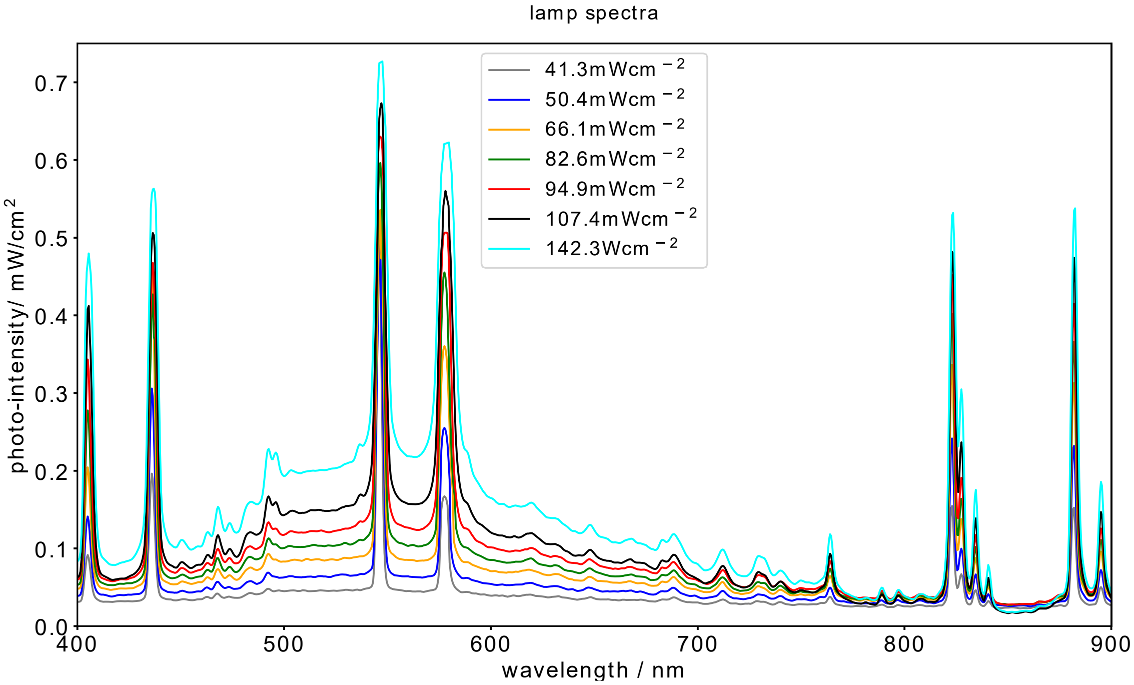

Supplementary Figure 15. Xenon-mercury arc lamp spectrum as a function of wavelengths, using a spectrometer with 0.2 nm wavelength interval at various incident photo-intensities, as measured separately with a photodetector. The incident photo-intensity values from the light source was measured with a photo-detector at the same distance of approximately 7cm between the front end of the coated rod and the light source.

Supplementary Discussion (Density Functional Theory simulations)

We first initialized the unit cell of In_2O_3 to generate a surface (111) plane interacting with the empty space of the unit cell box. The real surface of In_2O_3 comprises of many high index planes such as (211), (222) and (622). The predominant (222) plane can be decomposed into 2 atomic (100) and 2 atomic (011) planes. The unit cell of In_2O_3 (CIF file #2310009) was thus transformed using the TRANSTRU program to generate a surface (111) plane, and the transformed lattice structure does preserve its

periodicity as seen in its calculated XRD spectrum.

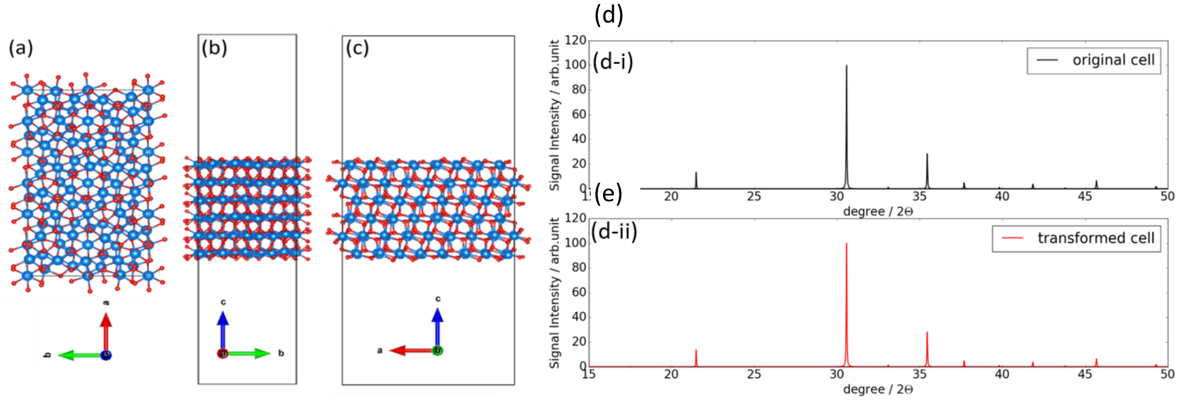

Supplementary Figure 16. (a-c) Initialized In_2O_3 (111) slab in the middle of the simulation box after transformation from the original CIF file. (d) Calculated XRD via Vesta between the original cell and In2O2 slab (d,e).

The DFT simulations (via Quantum Espresso) were implemented on an In_2O_3 surface with an oxygen vacancy and a hydroxyl group terminated on a neighboring Indium site to generate an active site of $In_2O_{3-x}(OH)_y$. The unit cell was relaxed with a plane wave kinetic energy cut-off of 48.6 Ry, pseudopotentials of pbe-rrkjus for Indium, Oxygen, Carbon and Hydrogen. The unit cell and the subsequent H_2 and CO_2 reactions relaxation was when the convergence point of 1×10^{-6} eV was achieved. Since the oxygen vacancy acts as an electron donor, the In-OH site remains stable after geometric relaxation. Adjacent to the oxygen vacancy and the In-OH is an under-coordinated Indium site that is unsaturated.

As the below sequence of reactions show, an incident H_2 interacts with the bent OH site and the under-coordinated and under-saturated indium sites, with one H atom interacting with the oxygen ion, and another interacting with the indium site (**1**). In order to neutralize the negative and positive charge of the OH and the In sites, heterolytic dissociation of H_2 occurs, with the formation of a negatively charged H on indium (-0.34q), and positive H (+0.39q) on the In-OH site (**2**). Continued geometric relaxation moves the H^+OH species away from the In-H^- site (**3**) with a shortening of the OH^+ bond to be a similar length as the other OH bond. When a CO_2 molecule approaches with the oxygen incident towards the hydrogenated site (**4**), CO_2 activation occurs due to the presence of the In-H^- interacting with the oxygen of CO_2 , and H^+OH interacting with the carbon of CO_2 . Bending of the CO_2 molecule and the temporary formation of a H^-CO_2 anion occurs (**5**), which is similar to $^{\cdot}CO_2^-$ radicals typically seen on other types of catalysts. Since the H^-CO_2 species has excess negative charge, it is neutralized by the of H^+OH and H_2O is formed from the CO_2 complex (**6**). Further relaxation stabilizes the CO and H_2O molecule, with a minor caveat that continued relaxation and movement of the H_2O molecule can interact with the under-coordinated Indium site and oxidizes the site.

Since the experimental results of the XPS measurements showed the initial presence of oxygen lattice, oxygen vacancy and OH species, it is possible for all three species to co-operate synergistically to set up a strong positive and negative charged complex for polarizing CO_2 molecule. The introduction of H_2

increases the formation of In-H^+OH as seen in the DFT results, with a significant positive binding energy shift, while the Indium shifts are relatively minor, indicating a compensating negative charge on the indium species. Furthermore, the XPS low binding energy spectra showed a defect associated tail, which is also manifested in the optical absorption spectrum. From the wavelength-CO action spectroscopy results, it can be seen that significant amount of CO is being produced at the visible wavelengths associated with these defects. Since hydroxyl/vacancies and unsaturated Indium defects are known to form respectively a large conduction band and valence band edge continuum of states within the optical band gap, it is thus likely that oxygen vacancies and OH work synergistically to activate H_2 and CO_2 to dissociate into CO and H_2O.

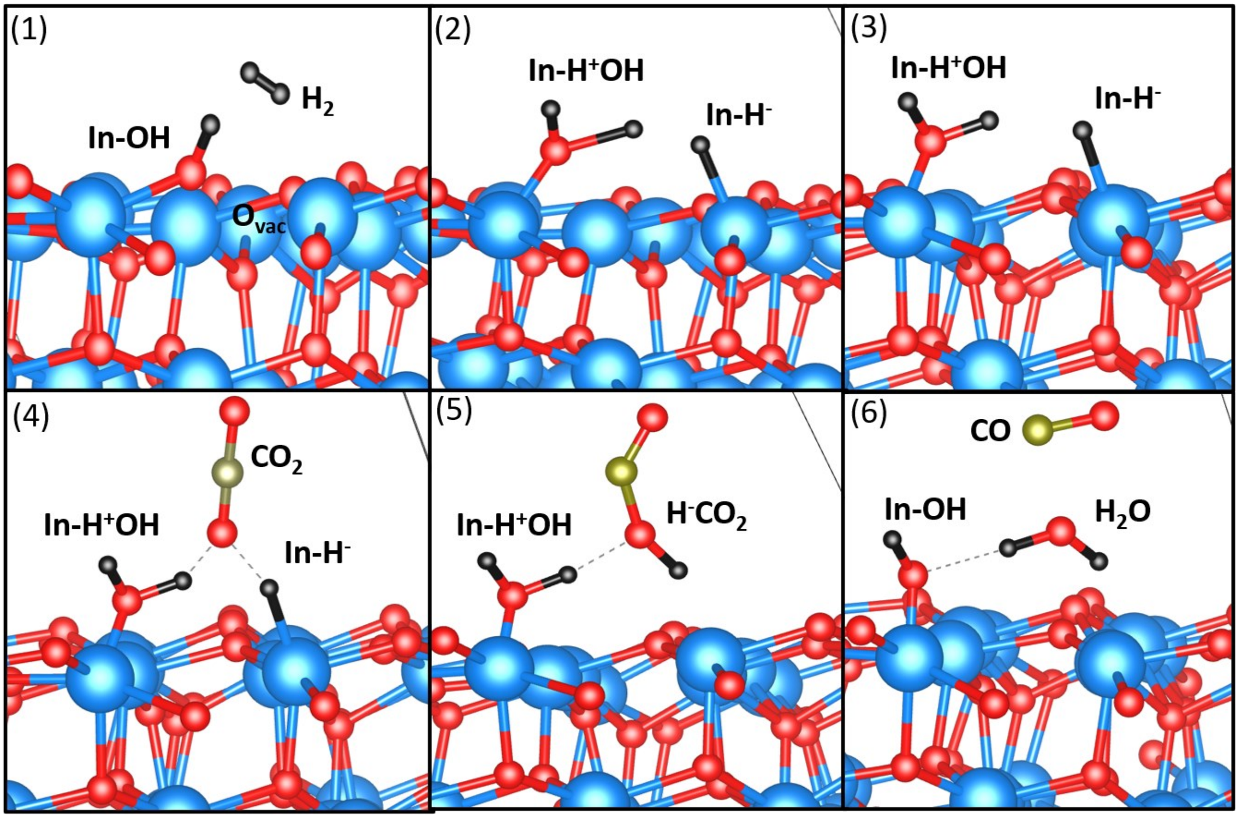

Supplementary Figure 17. (1-3) Sequence of H_2 dissociation on a OH terminated Indium site and a coordinately unsaturated Indium site. (4-6) Sequence of CO_2 dissociation on a H^+OH terminated Indium site and an Indium hydride site.

<u>Supplementary Note 2</u>

The aluminum foil itself produces 0.09 ppm yield of CO at 100°C under photo-illumination intensity of ~80mW/cm^2 after 1 hour of reaction time. Since the typical CO yield of the aluminum foil encapsulated $In_2O_{3-x}(OH)_y$ coated waveguide is 6.0-6.2 ppm within that same set of reactor parameters, the contribution of the aluminum foil itself is less than 1.5% of the total CO yield by the encapsulated $In_2O_{3-x}(OH)_y$ coated waveguide.
